# Long-Term Subjective Efficacy of Female Stress Urinary Incontinence with Tension-Free Vaginal Tape-Obturator Technique

**DOI:** 10.1007/s00192-024-05962-6

**Published:** 2024-11-04

**Authors:** Songwen Nian, Xiaoqing Wang, Ye Lu

**Affiliations:** Department of Obstetrics and Gynecology, Peking University First Hospital, No. 1 Xi’anmen Street, Beijing, 100034 China

**Keywords:** Long-term subjective efficacy, Quality of life, Stress urinary incontinence, TVT-O

## Abstract

**Introduction and Hypothesis:**

The objective was to evaluate the long-term subjective efficacy of the tension-free vaginal tape-obturator (TVT-O) technique in the treatment of female stress urinary incontinence (SUI).

**Methods:**

A retrospective analysis was performed on 84 patients who underwent TVT-O surgery for SUI in a tertiary center between January 2007 and December 2013. All patients filled in the Urinary Incontinence Quality of Life Questionnaire (I-QOL), the International Consultation on Incontinence Questionnaire Short Form (ICIQ-SF), and the Pelvic Organ Prolapse/Urinary Incontinence Sexual Questionnaire short form (PISQ-12). Subjective efficacy, including surgical efficacy, clinical severity of SUI, improvement in quality of life (QoL), and sexual activity with regard to TVT-O were compared before and after surgery (≥ 10 years).

**Results:**

The average postoperative follow-up time was 12.6 ± 1.9 years, range, 10–16 years. The overall subjective effectiveness of the surgery was 94.0% (79 out of 84). The subjective clinical severity significantly improved more than 10 years after surgery compared with the preoperative value (*p* = 0.000). The median I-QOL score was 88.1 (84.1–92.0) preoperatively and 98.3 (94.3–99.7) postoperatively, and the long-term QoL of postoperative patients was significantly improved (*p* < 0.05). The median ICIQ-SF score was 10.5 (8–15) preoperatively, and 3 (0–5) postoperatively, and the ICIQ-SF score before and after surgery showed significant improvement in urinary incontinence symptoms (*p* < 0.05). No difference was observed in the PISQ-12 scores before and after surgery in the sexually active population.

**Conclusions:**

The TVT-O technique still has good subjective efficacy in SUI more than 10 years after surgery and significantly improves the QoL of patients.

## Introduction

Stress urinary incontinence (SUI) is a common condition in middle-aged and older women, and the International Continence Society defines SUI as involuntary leakage of urine from the urethral opening during increased abdominal pressure, such as sneezing, coughing, laughing, or exercise [1]. The prevalence of SUI in adult Chinese women is as high as 18.9%, [2] which affects the quality of life (QoL) of patients to varying degrees. Currently, transvaginal midurethral tension-free sling surgery is the first-line surgical treatment for SUI [3, 4]. To effectively avoid the problem of bladder perforation of traditional surgery, in 2003 De Leval [5] invented a tension-free vaginal tape-obturator (TVT-O) punctured from the inside to the outside through the obturator hole, which provides stable sub-urethral support with a gentler subfascial hammock effect, simple operation, less trauma, and is currently widely used [6–8].

In our previous study [9] we showed that the objective cure rates of TVT-O were 85.1%, 86.2%, 86.5%, and 84.0% at 6 weeks, 3 months, 6 months, and 1 year after surgery, the total effective rate of surgery was 96.8%, 97.8%, 97.8%, and 97.3% respectively, and the subjective satisfaction rate (Incontinence Quality of Life [I-QOL] questionnaire) was 97.9% at 1 year after surgery. There are few relevant studies on whether the long-term efficacy more than 10 years after surgery can be as satisfactory as the short-term efficacy. Therefore, we summarized the data of patients > 10 years after TVT-O surgery at Peking University First Hospital to explore its long-term subjective efficacy.

## Materials and Methods

### Participants

This retrospective cohort study was conducted in a tertiary center in China. Patients were eligible if they had SUI and no contraindications to surgery. Patients were excluded if they could not tolerate surgery, were pregnant, or could not be followed up for various reasons. Data on the patients’ demographic characteristics were collected. We reviewed and analyzed a total of 120 patients who underwent TVT-O surgery for SUI between January 2007 and December 2013, of whom 84 had complete follow-up data, The specific demographic characteristics are shown in Table 1. All patients completed the I-QOL questionnaire [10], the International Consultation on Incontinence Questionnaire Short Form (ICIQ-SF) [11], and the Pelvic Organ Prolapse/Urinary Incontinence Sexual Questionnaire short form (PISQ-12) [12] before surgery and we contacted patients after surgery(≥ 10 years) to fill in questionnaires again by telephone interview.

**Table 1:** Demographic and perioperative data for the enrolled patients

Variables	Value
Age (years)	51 (47–63)
BMI (kg/m^2^)	24.8 (22.9–27.8)
Normal weight (18.5–23.9)	28 (33.3)
Overweight (24–27.9)	39 (46.4)
Obesity (≥ 28)	17 (20.2)
Gravidity	3 (2–4)
Parity	1 (1–2)
Menstruation	
Pre-menopause	45 (53.6)
Post-menopause	39 (46.4)
Delivery mode	
Vaginal delivery	78 (92.9)
Cesarean delivery	5 (6.0)
Both	1 (1.2)
History of hysterectomy	2 (2.4)
History of pelvic organ prolapse surgery	2 (2.4)
History of radical mastectomy	1 (1.2)
Complications	
Diabetes mellitus	9 (10.7)
Hypertension	25 (29.8)
Coronary heart disease	5 (6.0)
Asthma	1 (1.2)

Values are presented as *n* (%) or median (interquartile range)

*BMI* body mass index

This study was conducted in accordance with the Declaration of Helsinki. Given the retrospective nature of this study, informed consent from women included in the analysis was not required.

### Methods

Of these 84 patients, 15 underwent TVT-O surgery alone, and 69 underwent other surgeries. Seven patients underwent hysteroscopic surgery, 6 had laparoscopic cyst excision or adnexal excision or myomectomy, 3 had transabdominal hysterectomy, 3 had laparoscopically assisted vaginal hysterectomy, 6 had vaginal hysterectomy, and 44 underwent prolapse surgery. TVT-O adopts the Johnson & Johnson Medical Co., Ltd. box set device; the specific surgical method is described in the literature [5]. The catheter was indwelling for 3 (2–5) days and then removed.

### Postoperative Follow-Up Outcomes

Perioperative details, including operative time, blood loss volume, and complications, were recorded. The mean follow-up time after surgery was 12.6 ± 1.9 years, ranging from 10 to 16 years.

The primary outcome was subjective efficacy, including surgical efficacy, clinical severity of SUI, and improvement in QoL. Surgical efficacy included subjective cure/improvement/failure [2]. Subjective cure was defined as no urine leakage in the case of increased abdominal pressure such as coughing. Subjective improvement was defined as urinary leakage in the case of increased abdominal pressure such as coughing, but the frequency or amount of urinary leakage was reduced compared with that before surgery. Ineffectiveness was subjectively considered when the frequency or amount of urine leakage was not significantly reduced or even aggravated compared with that before surgery. The clinical severity of SUI is based on the Ingelman-Sundberg scoring method (subjective) [2, 13]. Mild SUI was defined as urinary incontinence (UI) that occurs when coughing and sneezing without using a pad. Moderate SUI was defined as UI that occurs during daily activities such as running, jumping, and brisk walking, and requires the use of a urine pad. Severe SUI was defined as UI with light activity and a change in supine position. Two questionnaires were used to estimate the improvement in QoL. The I-QOL questionnaire was developed by Wagner et al. [10] in 1996 to assess the QoL of patients with SUI, including a total of 22 short questions about avoidance and limiting behaviors, psychosocial impacts, and social embarrassment. The ICIQ-SF questionnaire developed by Hay-Smith et al. [11] was used to assess the severity of UI in patients and its impact on daily life, which is economical, convenient, and practical. It comprises three questions regarding the frequency, severity, and QoL impact of UI; the higher the score, the more serious the SUI.

Secondary outcomes included late complications and sexual function. The former included urinary frequency and/or urgency, difficulty urinating, postoperative pain, tape erosion, and/or extrusion [14]. We evaluated postoperative pain by asking patients if they were experiencing groin or thigh pain, vulvar and vaginal pain. When we assessed tape exposure, we first asked patients about the common symptoms, such as vaginal bleeding, vaginal discharge, and pain in sexual partners during intercourse; then, we instructed patients to touch the anterior vaginal wall 3 cm proximal to the vaginal orifice, and ask them if they had a sensation of a foreign body or tingling. There were 3 patients who felt that vaginal secretion had increased and came to the hospital for examination, but our examination revealed no tape exposure. The PISQ-12 was validated in the Chinese language and used to measure sexual activity in relation to TVT-O, with higher scores indicating better sexual function [15].

### Statistical Analysis

Continuous variables were reported as mean ± standard deviation when the variables were normally distributed or were approximately normally distributed, and non-normally distributed variables were expressed as medians (interquartile ranges). Categorical variables are reported as numbers and percentages (%). In the primary analysis, surgical efficacy was reported as descriptive (frequency). The Wilcoxon signed-rank test was used to compare the preoperative and postoperative clinical severity of the SUI, I-QOL, and ICIQ-SF scores. Differences were considered statistically significant when the *p* value was < 0.05. Statistical analyses were performed using SPSS version 24.

## Results

All surgeries were completed. The average operation time for TVT-O alone was 23.9 min and the intraoperative blood loss was 5–50 ml. There were no perioperative complications, such as vascular or bladder/vaginal injury, hematoma, or infection. After the urinary catheter was removed, 2 patients (2.4%) had urinary retention, and the urinary catheter was re-indwelled for 3 days. After the catheter was removed again, the patient could urinate smoothly, and the residual urine volume was measured at < 50 ml. Seven patients (8.3%) had postoperative pain; in 1 patient the pain was relieved after physiotherapy, but the remaining 6 cases had no special treatment, and the symptoms were relieved within 1 week after surgery.

### Subjective Efficacy

In terms of surgical efficacy, subjective cure was observed in 37 patients (44.0%), improved in 42 patients (50.0%), and ineffective in 5 patients (6.0%). The efficacy rate was 94.0% (79 out of 84). Among the patients in whom treatment was subjectively ineffective, in 1 patient treatment was ineffective in the short term after surgery, and in 4 patients treatment was effective in the short term but not in the long term after surgery.

According to the Ingelman-Sundberg score (subjective), we found a significant improvement in the subjective clinical severity of SUI more than 10 years after surgery compared with the preoperative value (*p* = 0.000), which is shown in Table 2.

**Table 2:** Preoperative and more than 10 years postoperative clinical severity of stress urinary incontinence (SUI)

Subjective grading	Preoperatively	More than 10 years postoperatively	*Z*	*p*
Mild SUI	7 (7.9)	35 (41.7)	−7.486*	0.000
Moderate SUI	66 (78.6)	11 (13.1)		
Severe SUI	11 (13.1)	1 (1.2)		
Asymptomatic	0 (0.0)	37 (44.0)		

Data are presented as *n* (%)

*Wilcoxon signed-rank test

The quality of life of patients was evaluated using the I-QOL questionnaire, and the results showed that the QoL of patients more than 10 years after surgery improved in terms of avoidance and limiting behaviors, psychosocial impacts, and social embarrassment compared with preoperative years, and the difference was statistically significant before and after surgery (*p* < 0.05). The long-term QoL of patients after surgery significantly improved (Table 3).

**Table 3:** Preoperative and more than 10 years postoperative quality of life

Questionnaire score	Preoperatively	More than 10 years postoperatively	Z*	*p*
I-QOL	88.1 (84.1–92.0)	98.3 (94.3–99.7)	−6.570	0.000
Avoidance and limiting behaviors	37 (34–38)	39 (37–40)	−4.899	0.000
Psychosocial impacts	42 (40–43)	45 (44–45)	−6.610	0.000
Social embarrassment	22 (21–23)	25 (24–25)	−6.398	0.000
ICIQ-SF	10.5 (8–15)	3 (0–5)	−7.445	0.000
UI frequency	3 (2–3)	1 (0–1)	−6.851	0.000
UI severity	2 (2–4)	2 (0–2)	−6.209	0.000
QoL impact of the UI	5 (3–10)	0 (0–2)	−7.387	0.000

Data are presented as median (interquartile range)

*I-QOL* Urinary Incontinence Quality of Life, *ICIQ-SF* International Consultation on Incontinence Questionnaire Short Form, *UI* urinary incontinence, *QoL* quality of life

*Wilcoxon signed-rank test

The ICIQ-SF was used to assess the severity of UI. The results showed that the severity of UI in patients decreased in terms of the frequency of urine leakage, the severity of urine leakage, and the degree of impact on QoL more than 10 years after surgery. The comparison of ICIQ-SF scores before and after surgery showed that the symptoms of UI after surgery were significantly improved compared with before, which was statistically significant (*p* < 0.05; Table 3).

### Late Complications

Urinary frequency and urgency were the most common long-term complications in this study. There was 1 case (1.2%) of new-onset urinary frequency that occurred 13 years after surgery and 10 cases (11.9%) of new-onset urinary urgency, of which 3 cases occurred 10 years after the operation, 2 cases occurred 11 years after the operation, 4 cases occurred 12 years after the operation, and 1 case occurred 13 years after the operation. Two patients (2.4%) had difficulty urinating (but could empty the bladder) after surgery; 1 case occurred 13 years after surgery, and 1 case occurred 10 years after surgery. No long-term postoperative pain, tape erosion, or extrusion was observed.

### Sexual Activity

Of these 84 patients, 17 were sexually active more than 10 years after surgery, and only 15 patients completed the PISQ-12 because 2 patients refused to have a sexual life assessment. The median preoperative PISQ-12 score was 12 (10, 16), and 14 (11, 17) postoperatively, and the PISQ-12 score before and after surgery did not differ significantly (*p* = 0.789).

## Discussion

With the improvement in people's health awareness and QoL requirements, the impact of SUI on QoL has also received increasing attention, especially because an increasing number of young patients choose surgery to treat SUI. It is essential to evaluate the long-term efficacy of TVT-O in patients to improve their long-term QoL and to weigh the risks and benefits of reoperation.

Relatively few studies have investigated the long-term efficacy of TVT-O after surgery. The postoperative follow-up time for TVT-O in this study was 10–16 years, and the subjective effective rate after surgery was 94.0% (79 out of 84). Serati et al. [16, 17] conducted a multicenter prospective study and found that the subjective cure rate of patients with SUI who underwent TVT-O surgery at 13 years after surgery was 95% (150 out of 157), at 10 years it was 97% (155 out of 160), and there were no significant differences compared with 5 years (95%, 155 out of 161) or 1 year (95%, 157 out of 165) after surgery. One cohort study with an average follow-up of 9 years [18] showed a subjective cure rate of 85% (90 out of 106), who considered subjective cure when the patient answered that they were very satisfied or satisfied with the surgery. A 12-year prospective follow-up study by Zhang et al. [19] showed that the subjective satisfaction rate (overall impression of improvement) of 73 patients who underwent TVT-O surgery was 80.8%. Our previous study [9] showed that the total effective rate of TVT-O 1 year after surgery was 97.3%, and the total effective rate more than 10 years after surgery in this study was 94.0%, which was slightly lower than 1 year after surgery, but the overall long-term efficacy was still certain; the reason for the decrease in the rate of effectiveness may be that with the extension of time, the patient's age is further increased, the pelvic floor tissue may be further relaxed and aging, and some patients may have symptoms of urine leakage during mild exertion. Moreover, some patients mistook symptoms of overactive bladder, such as frequent urination and urgency due to bladder aging, for a recurrence of SUI. Of course, some patients had loose slings owing to increased abdominal pressure for many years, and the symptoms recurred.

Our study found improvements in SUI-related symptoms and QoL after TVT-O for more than 10 years. Various studies [20–23] have shown improvement, but follow-up for more than 10 years after surgery is rare. Sun et al. [24] used the Incontinence Impact Questionnaire Short Form to evaluate the QoL of patients with SUI and found that it was still significantly improved 10 years after TVT-O surgery. In UI symptoms, a 4-year Spanish cohort study [21] found a significant difference in ICIQ-SF scores before and after TVT-O surgery in patients with SUI (*p* < 0.05). In a multicenter prospective study [17] that followed 157 SUI patients undergoing TVT-O surgery for up to 13 years, ICIQ-SF scores showed significant improvement in long-term UI symptoms after TVT-O surgery (*p* < 0.01). TVT-O improves the long-term QoL of patients.

Complications after TVT-O include postoperative pain, voiding disorders, new overactive bladder, tape erosion and/or extrusion, and are rare [14]. New-onset urinary frequency and/or urgency were the most common long-term complications after surgery, with an incidence rate of 13.1% (11 out of 84) in this group. It cannot be ruled out that patients may have mild urge UI before surgery; after the improvement of SUI symptoms, the symptoms of urge incontinence became more prominent, or the sling was slightly tight. Foreign body irritation, increasing age [25], urinary tract infections, and other factors may be related to the occurrence of symptoms. We were required to accurately grasp the surgical indications, communicate with the patient, and explain the procedure before surgery. Attention should be paid not to place the sling too tightly during surgery. This study shows that TVT-O has fewer long-term complications overall, has little impact on life, and is relatively safe.

The main strength of this study was that the postoperative follow-up time was very long, and we used surgical efficacy, the clinical severity of SUI, and two authoritative questionnaires to comprehensively estimate the subjective surgical efficacy of SUI. However, our research on sexual activity is not satisfactory owing to the small number of people who were still sexually active in the long term after surgery and were willing to participate in the sexuality survey.

In conclusion, the long-term subjective efficacy of TVT-O in the treatment of SUI in women is certain, which significantly improves the QoL of patients and has the characteristics of few intraoperative and postoperative complications, making it a safe and effective operation. However, its effects on sexual life need to be studied further.

## Data Availability

The data that support this study are available from the corresponding author upon reasonable request.

## Abbreviations

BMI: Body mass index
ICIQ-SF: International Consultation on Incontinence Questionnaire Short Form
I-QOL: Incontinence Quality of Life
PISQ-12: Pelvic Organ Prolapse/Urinary Incontinence Sexual Questionnaire short form
QoL: Quality of life
SUI: Stress urinary incontinence
TVT-O: Tension-free vaginal tape-obturator
UI: Urinary incontinence
